# Bioactive silver nanoparticles fabricated using *Lasiurus scindicus* and *Panicum turgidum* seed extracts: anticancer and antibacterial efficiency

**DOI:** 10.1038/s41598-024-54449-3

**Published:** 2024-02-20

**Authors:** Najla Alburae, Rahma Alshamrani, Afrah E. Mohammed

**Affiliations:** 1 Department of Biological Sciences, King Abdulaziz University, P.O.BOX 80206, 21589 Jeddah, Saudi Arabia; 2 Department of Biology, College of Science, Princess Nourah bint Abdulrahman University, P.O. Box 84428, 11671 Riyadh, Saudi Arabia

**Keywords:** Bio nanotechnology, Biomedicine, Ultrastructural changes, Cell damage, Biotechnology, Cell biology

## Abstract

Applying extracts from plants is considered a safe approach in biomedicine and bio-nanotechnology. The present report is considered the first study that evaluated the seeds of *Lasiurus scindicus* and *Panicum turgidum* as biogenic agents in the synthesis of silver nanoparticles (AgNPs) which had bioactivity against cancer cells and bacteria. Assessment of NPs activity against varied cell lines (colorectal cancer HCT116 and breast cancer MDA MBA 231 and MCF 10A used as control) was performed beside the antibacterial efficiency. Different techniques (DLS, TEM, EDX and FTIR) were applied to characterize the biosynthesized AgNPs. The phytochemicals from both *L. scindicus* and *Panicum turgidum* were identified by GC–MS analysis. Spherical monodisperse NPs at average diameters of 149.6 and 100.4 nm were obtained from seed extract of *L. scindicus* (L-AgNPs) and *P. turgidum*, (P-AgNPs) respectively. A strong absorption peak at 3 keV is observed by the EDX spectrum in the tested NPs. Our study provided effective NPs in mitigating the tested cell lines and the lowest IC_50_ were 7.8 and 10.30 for MDA MB231 treated by L-AgNPs and P-AgNPs, respectively. Both fabricated NPs might differentially target the MDA MB231 cells compared to HCT116 and MCF10A. Ultrastructural changes and damage for the NPs-treated MDA MB231 cells were studied using TEM and LSM analysis. Antibacterial activity was also observed. About 200 compounds were identified in *L. scindicus* and *P. turgidum* by GC–MS analysis might be responsible for the NPs reduction and capping abilities. Efficient NPs against cancer cells and microbes were obtained, however large-scale screening is needed to validate our findings.

## Introduction

*Lasuirus scindicus* is C4 perennial grass belonging to the Poaceae family that is known as nutritiously valuable and hard environment tolerant grass^1,2^. Dry regions of Saudi Arabia are one natural source of *L. scindicus*. Antioxidant activity and presence of tannin, saponins, flavonoids, alkaloids and total phenolics in *L. scindicus* were stated by Al-Rwaily et al.^2^. Further, *Panicum turgidum* is also a perennial grass of the Poaceae family well spread in deserts and salty areas and can be used as a maize alternative in cattle feedings^3^. *P. turgidum* revealed safe antihepatotoxic activity and about 39 metabolites that have been identified using UPLC coupled to qTOF-MS analysis where C-flavonoids as the major constituents^4^. The two described plants could tolerate harsh conditions such as deserts therefore, their richness in unique secondary metabolites is expected to enhance their stress tolerance ability. Secondary metabolites are known as good agents in biomedicine and nanotechnology therefore seeds of such two grasses were chosen for the current study. Plant active ingredients can be good candidates for the biological activity of plants besides nanoparticles (NPs) formulation since are known as reducing and capping agents. The importance of *L. scindicus* as a medicinal plant was earlier studied and the antifungal and antibacterial abilities of *P. turgidum* were reported^5,6^, however, no valid report about their anticancer ability and their ability in NPs fabrication.

Nanotechnology and nanomedicine are gaining great concern due to NPs' unique characteristics that have varied biological activities such as antibacterial and anticancer abilities^7^. Silver nanoparticles (AgNPs) are known as potential nano antibiotics due to their antimicrobial ability^8^ that overcome microbial resistance to conventional ones^9^. The AgNPs in cancer mitigation were also a well-known strategy that enhances mammalian cell death^10^ and therefore recommended as potential therapeutic anticancer drugs. AgNPs may cause cellular metabolic disruption^11^ or molecular damage^12^ when entering the cell via membrane disruption, such cellular changes could be the main mechanisms for NPs' biological activities besides the production of reactive oxygen species^13^. One more advantage for AgNPs is their fabrication by eco-friendly and feasible approaches using biogenic agents such as plant extract or other biological materials. Varied studies indicated the biosynthesis of AgNPs applying extracts from different biogenic agents such as plants, microbes, lichens and fungi^14–18^ and their biological activities were reported. Biomolecules from biogenic agents can be taken by the AgNPs, cover their surface and stabilize them from agglomeration^19^ therefore, controlled growth and development is expected for biogenic NPs. High compatibility with the biological system is expected for AgNPs capped with biomolecules^20^ which enhance their utilization in medical treatments^21^. The advantages of biogenic AgNPs encourage us to use rarely studied plants for their ability in NPs biosynthesis and biomedicine, therefore *L. scindicus* and *P. turgidum* seed extracts were examined and the prepared NPs were tested as anticancer and antibacterial agents. Their Mode of action against cancer cells was noted using TEM and LSM analysis. Before, the bio-fabricated NPs have been described utilizing different techniques such as TEM, DLS, and EDX analysis as well as the FTIR which was used for functional group detection.

## Results

### NPs characterization

**Figure 1:**
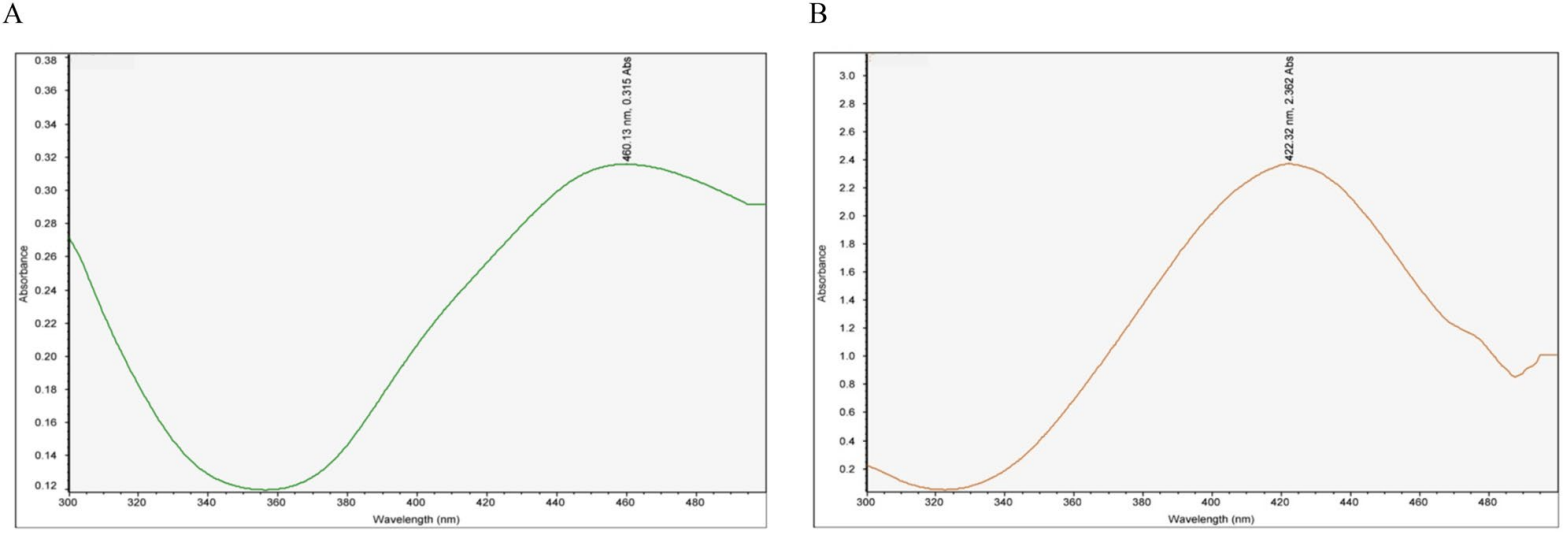
UV spectroscopy of L-AgNPs (**A**) and P-AgNPs (**B**).

The present research investigated AgNPs fabricated by two biogenic materials, the seeds of *Lasiurus scindicus* and *Panicum turgidum* which provided L-AgNPs and P-AgNPs, respectively. The full biotransformation of Ag^+^ ions from AgNO_3_ into Ag^0^ was noted when the color of the silver salt and seed extract was turned to dark brown after 6 h where the color intensity was increasing with time. The NPs solutions were scanned using wavelength scale (300 and 500 nm) where the maximum absorbances were observed at 460.13 and 422.32 nm for L-AgNPs and P-AgNPs, respectively (Fig. 1).

**Figure 2:**
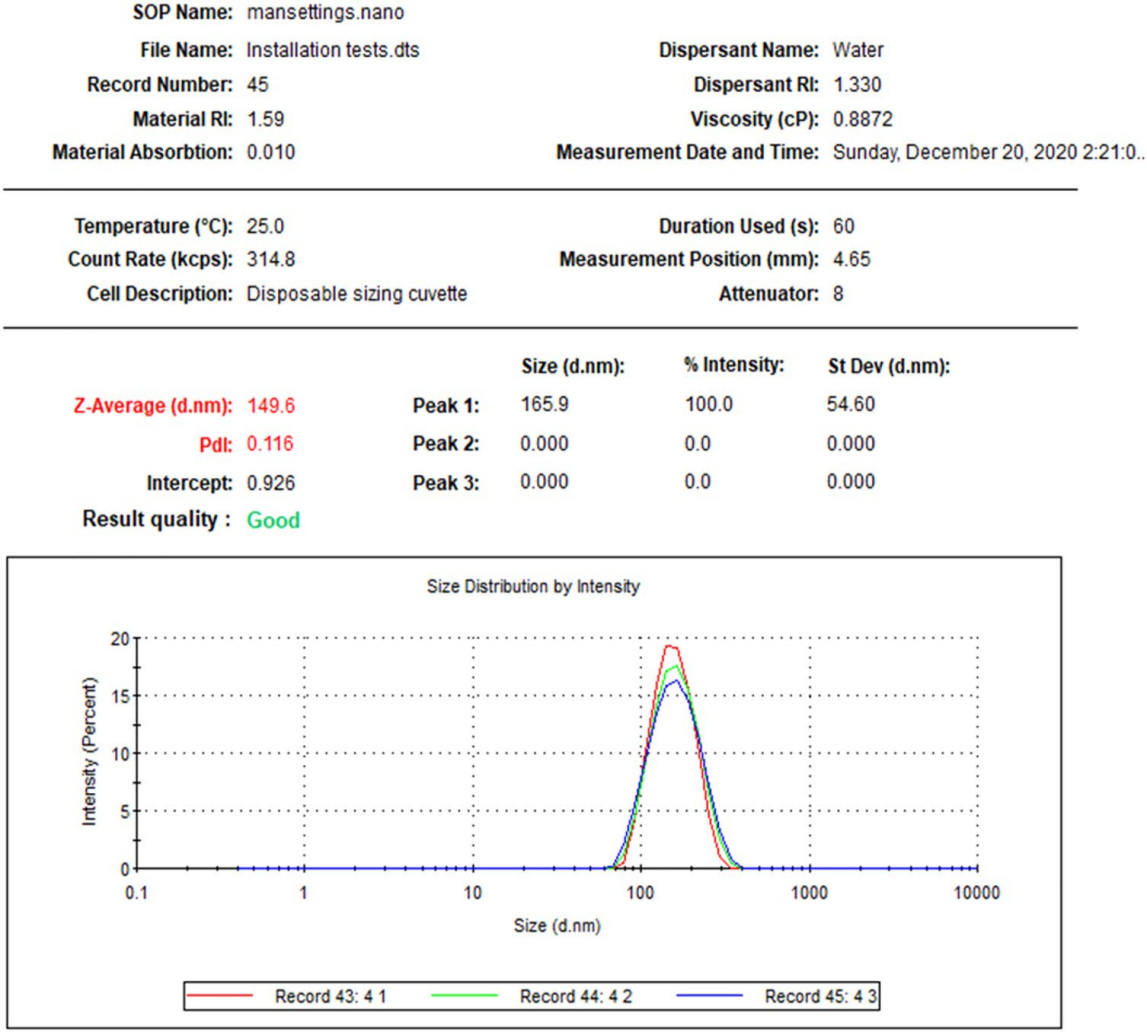
The mean size distribution of L-AgNPs for three readings that indicated by different colors.

**Figure 3:**
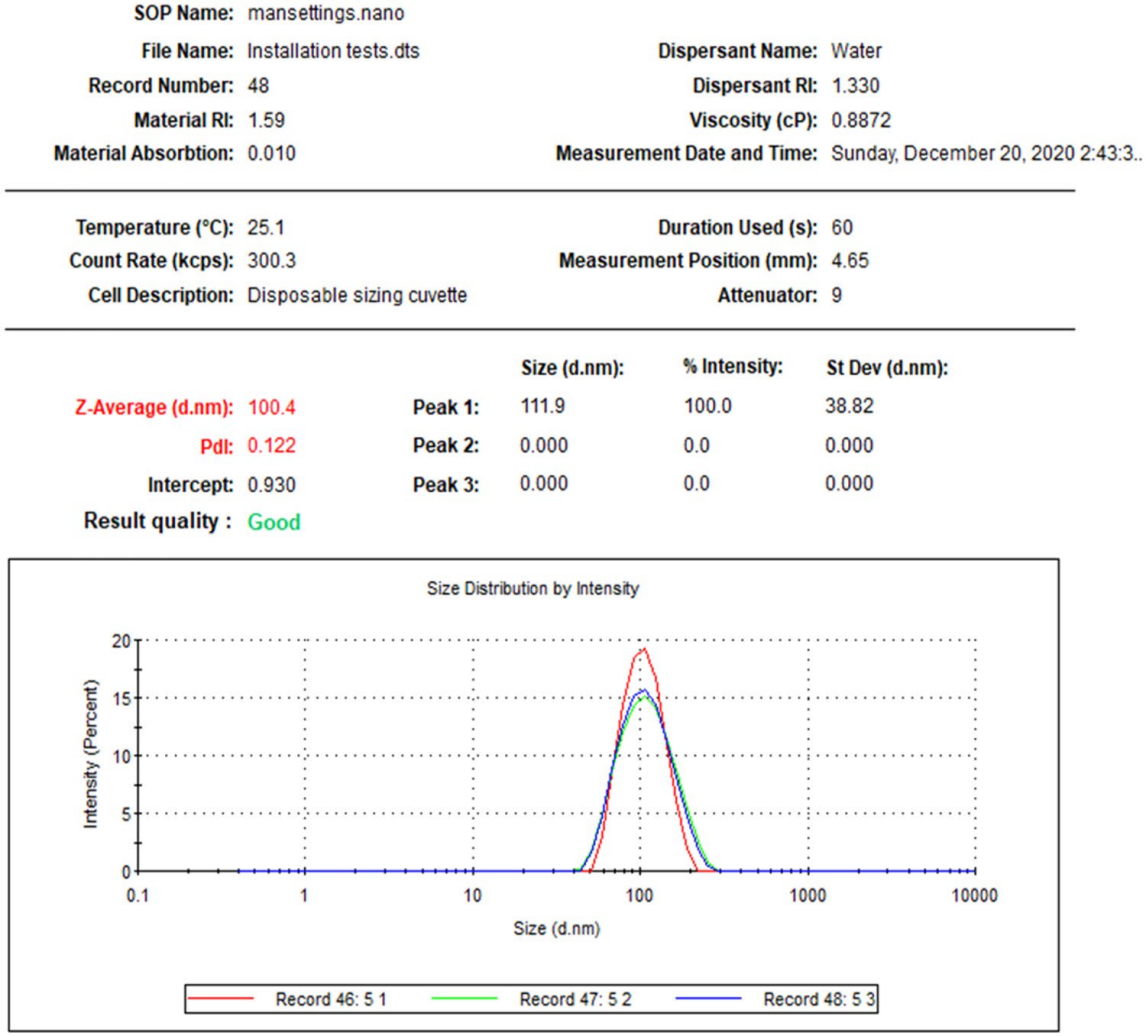
The mean size distribution of P-AgNPs for three readings that indicated by different colors.

As a second step, AgNPs have been separated for further analysis such as size distribution (Figs. 2 and 3) that displayed 149.6 and 100.4 nm as average diameter for L-AgNPs and P-AgNPs, respectively. polydispersity indices (PDI) are known as an essential factor that may affect the characteristics of fabricated NPs. Our findings indicated 0.116 and 0.112 as PDI for L-AgNPs and P-AgNPs, respectively.

**Figure 4:**
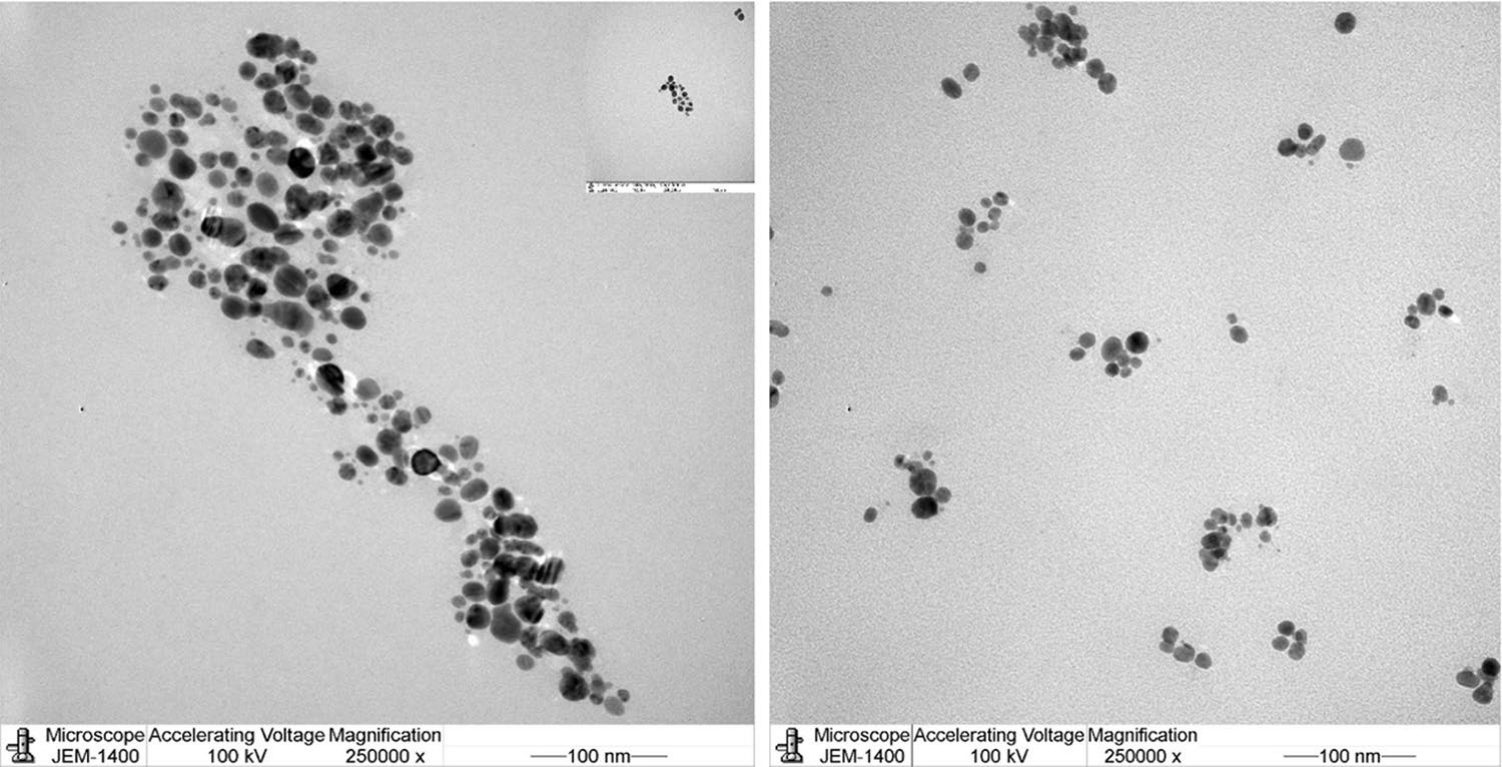
Frequency distribution of spherical shape L-AgNPs under TEM micrographs at a magnification of 250,000 and 100 nm scale bars.

**Figure 5:**
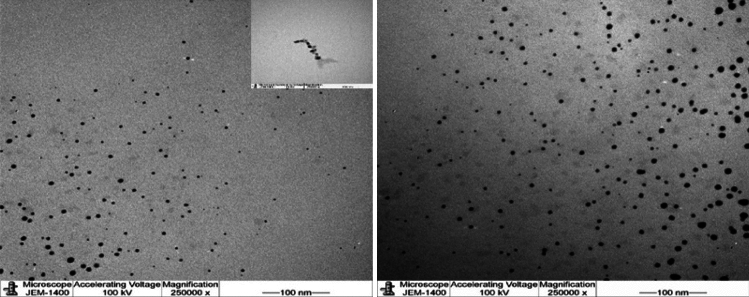
Frequency distribution of spherical shape P-AgNPs under TEM micrographs at a magnification of 250,000 and 100 nm scale bars.

**Figure 6:**
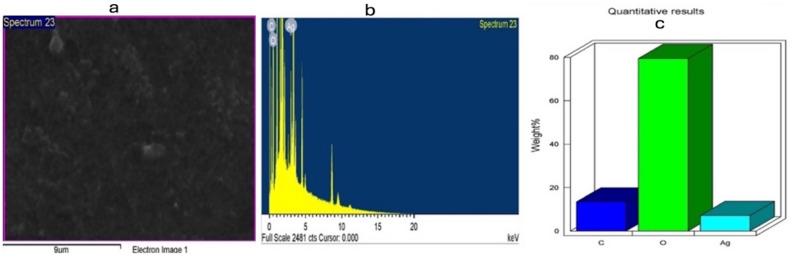
The morphology of L-AgNPs surface (**a**) and quantitative analysis of image using EDS for carbon, oxygen and silver atoms weights (**b**) and (**c**).

**Figure 7:**
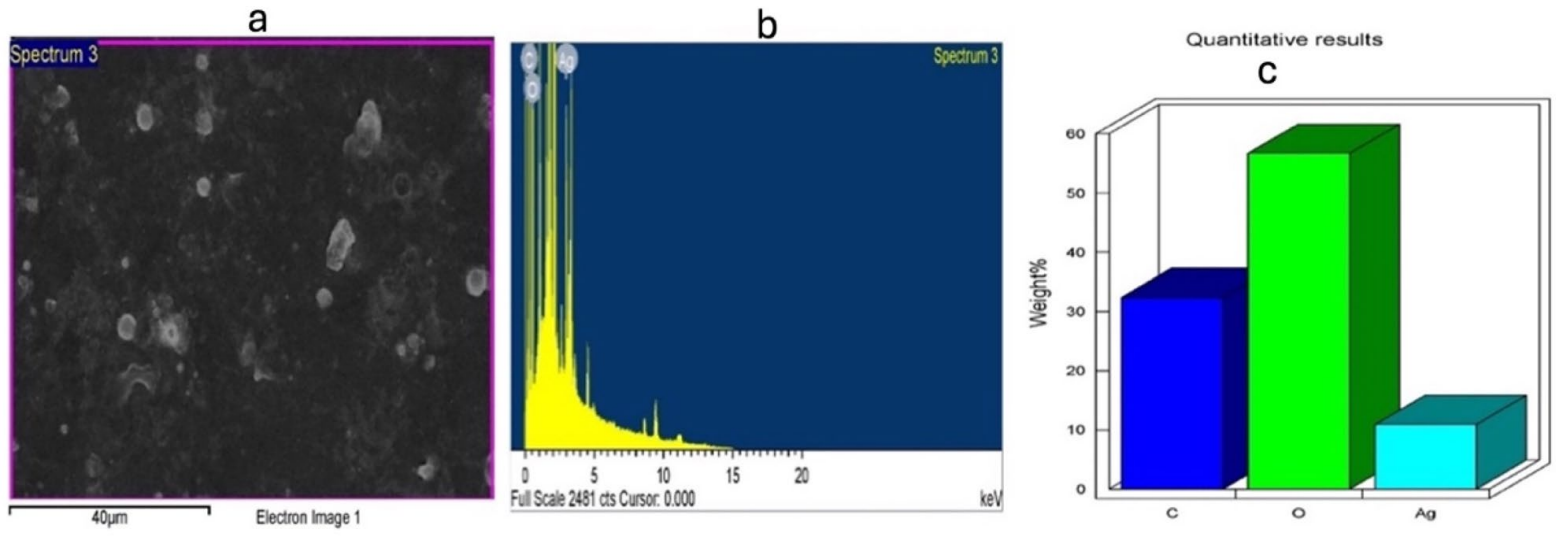
The morphology of P-AgNPs surface (**a**) and quantitative analysis of image using EDS for carbon, oxygen and silver atoms weights (**b**) and (**c**).

Further characterization was observed using TEM analysis as shown in Figs. 4 and 5 which displayed spherical shapes AgNPs with good dispersion. Mostly, no agglomeration was noted by TEM analysis. The EDX results are displayed in Figs. 6 and 7 for L-AgNPs and P-AgNPs, respectively. Results confirmed the generation of AgNPs following the individual incubation of each seed extract and Ag^+^. A strong absorption peak at 3 keV is observed by the EDX spectrum that is linked to the occurrence of elemental Ag in the nanoparticle’s solution with another two signals for carbon and oxygen.

### FTIR analysis

**Figure 8:**
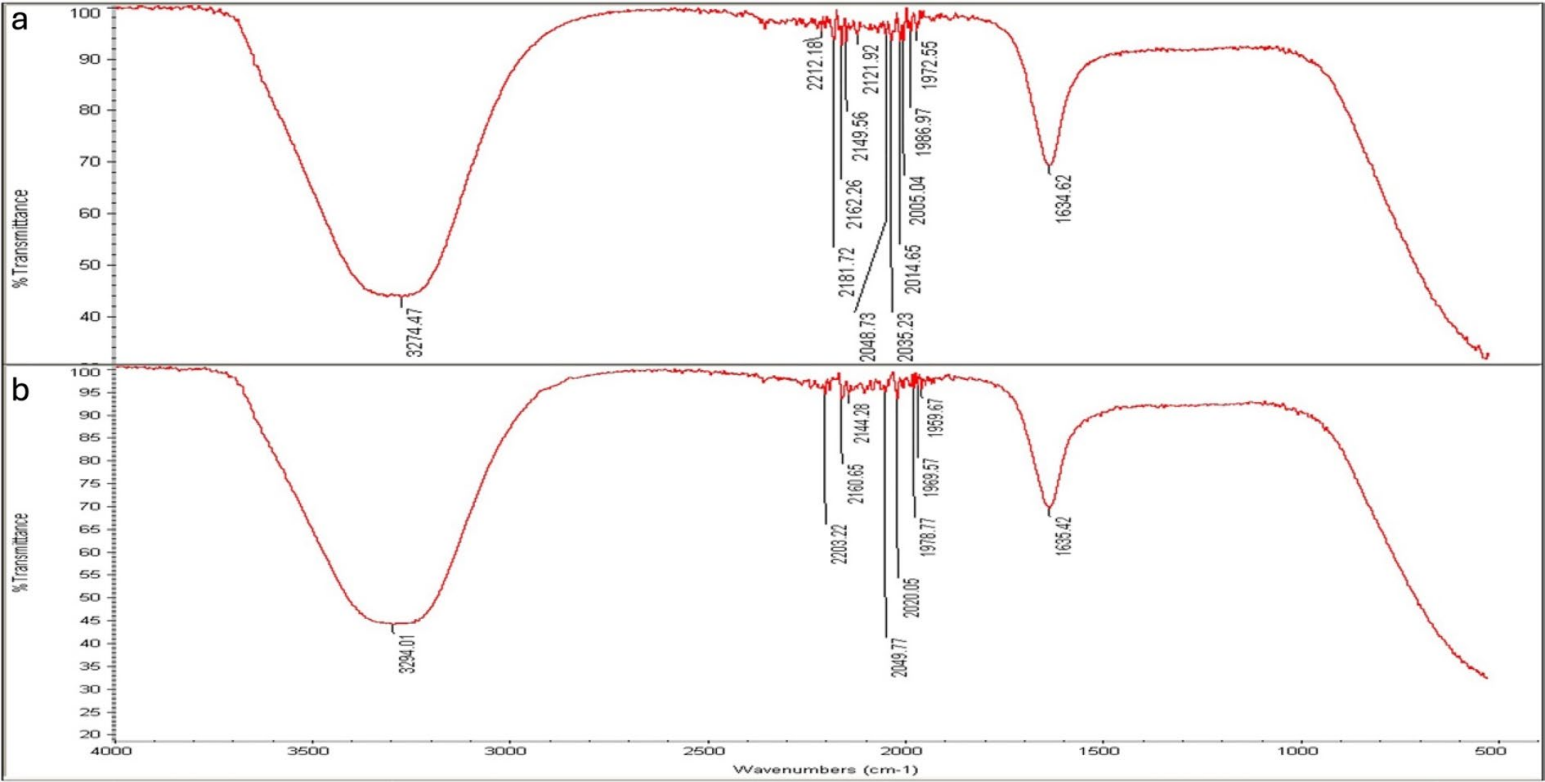
Absorbance peaks presenting the organic materials by FTIR analysis indicating *Lasiurus scindicus* seed extract (**a**) and NPs prepared by their aid (L-AgNPs) (**b**).

**Figure 9:**
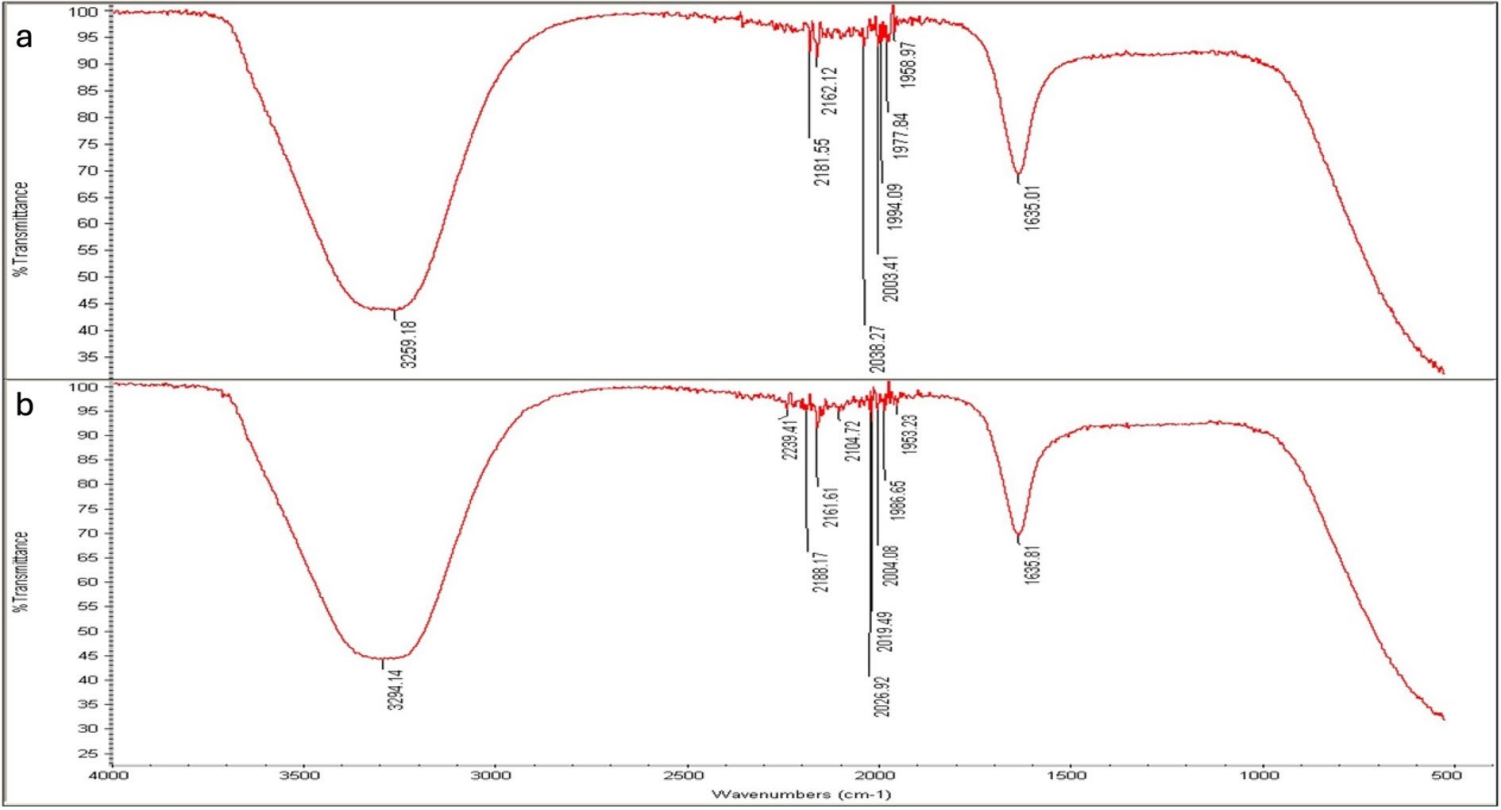
Absorbance peaks presenting the organic materials by FTIR analysis indicating *Panicum turgidum* seed extract (**a**) and NPs prepared by their aid (P-AgNPs) (**b**).

The role of different functional groups in AgNPs fabrication and bio-reduction process is presented by FTIR analysis when seed extracts and NPs were tested. High absorbance peaks at 3274.47 and 1634.62 cm^−1^ were noted for the seed extract of *L. scindicus* and 3294.01 and 1635.42 cm^−1^ for L-AgNPs (Fig. 8). Peaks at 3259.18 and 1635.01 cm^−1^ were noted for the seed extract of *P. turgidum* and 3294.14 and 1635.81 cm^−1^ for P-AgNPs (Fig. 9).

### Cytotoxic effect

**Figure 10:**
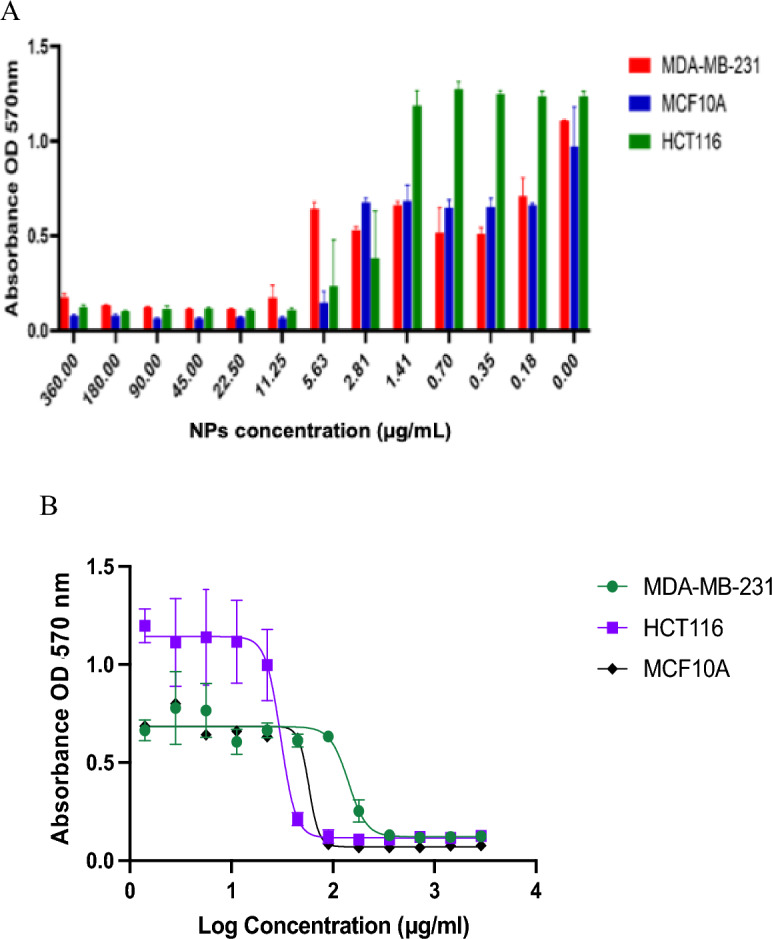
Consequences of L-AgNPs treatment on the cancer and normal cell viability. (**A**) presents the bar graph that indicates the dose–response of L-AgNPs on a normal cell line; MCF 10A and two human cancer cell lines; MDA MB 231 and HCT116 viability. (**B**) displays the effect of Log concentration of biogenic NPs against the normalized cell viability of one normal cell line MCF 10A and two human cancer cell lines; MDA MB 231 and HCT116.

**Figure 11:**
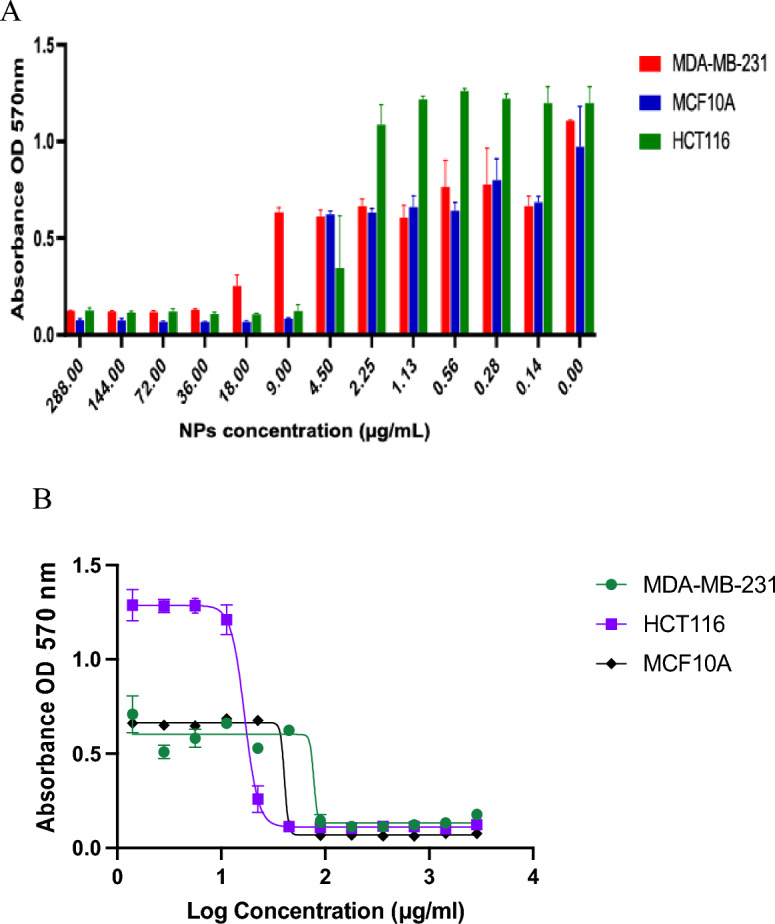
Consequences of P-AgNPs treatment on the cancer and normal cell viability. (**A**) presents the bar graph that indicates the dose–response of P-AgNPs on a normal cell line; MCF 10A and two human cancer cell lines; MDA MB 231 and HCT116 viability. (**B**) displays the effect of Log concentration of biogenic NPs against the normalized cell viability of one normal cell line MCF 10A and two human cancer cell lines; MDA MB 231 and HCT116.

The metabolic activity of human cell lines treated by L-AgNPs and P-AgNPs have been identified by the MTT test; one normal cell line (MCF 10A) and two cancer cell lines (HCT116 and MDA MB 231) as presented in Figs. 10 and 11. The preliminary assessment was designed by subjecting tested cell lines to varied concentrations of NPs at the range of 0–360 μg/mL for L-AgNPs and 0–288 μg/mL for P-AgNPs for 48 h. Clear induction of a dose-dependent manner in cell viability (*p* < 0.0001) was noted where the cell viability of NPs-treated cell lines was significantly decreased with dose increment. The IC_50_ values were quantified using the GraphPad Prism by fitting the curve of the cell viability to be 141.6 and 30.58 μg/mL for MDA MB231 and HCT116 and 57.97 for the normal cell line MCF10A treated by L-AgNPs. 77.59, 16.82 and 40.63 μg/mL were the IC_50_ for MDA MB231, HCT116 and MCF10A treated by P-AgNPs, respectively as shown in Table 1. Our findings indicated effective NPs in mitigating the HCT116 cancer cell and the nonmalignant epithelial human breast cell line which was used as a control. Both fabricated NPs might differentially target the colorectal cancer cell line tested since their concentration that needed to suppress 50% of the cells was lower compared to that needed for breast cancer and normal cell line suppression. The differential cytotoxic effect on breast cancer cell lines was weak compared to normal breast epithelial cells, therefore our NPs could not be recommended for MCF10A since the main reason that restricts the NPs for cancer mitigation is the expected toxicity against normal cell lines However, our studies confirmed high cytotoxic properties of L-AgNPs and P-AgNPs against the aggressive colorectal cancer cell lines however, the weak effect was noted against the aggressive breast cancer cell lines.

**Table 1:** IC_50_ (µg/ml) of biogenic NPs on MDA-MB-231, HCT116 and MCF10A cell lines.

Treatment	Cancer cell		Normal cell
	MDA-MB-231	HCT116	MCF10A
L-AgNPs	141.6	30.58	57.97
P-AgNPs	77.59	16.82	40.63

**Figure 12:**
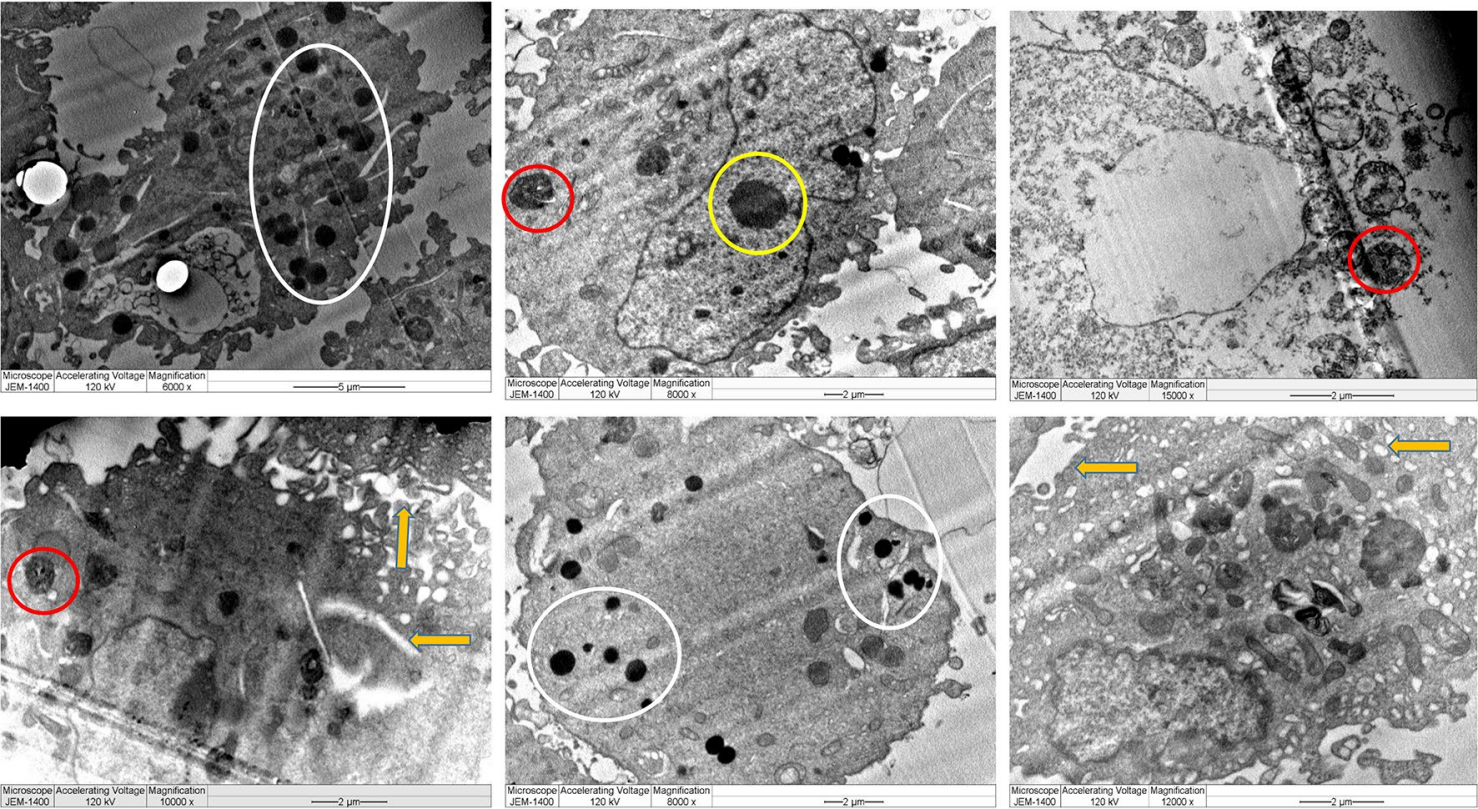
TEM analysis for the human cancer cell lines (MDA MB 23) treated with L-AgNPs at varied magnification showing ultrastructural alteration displaying chromatin condensation (yellow circle) and over whole cell shrinkage as well as damaged mitochondria (red circle), large vacuoles (yellow arrow) and peroxisome (white circle). Damaged cancerous cells are observed.

**Figure 13:**
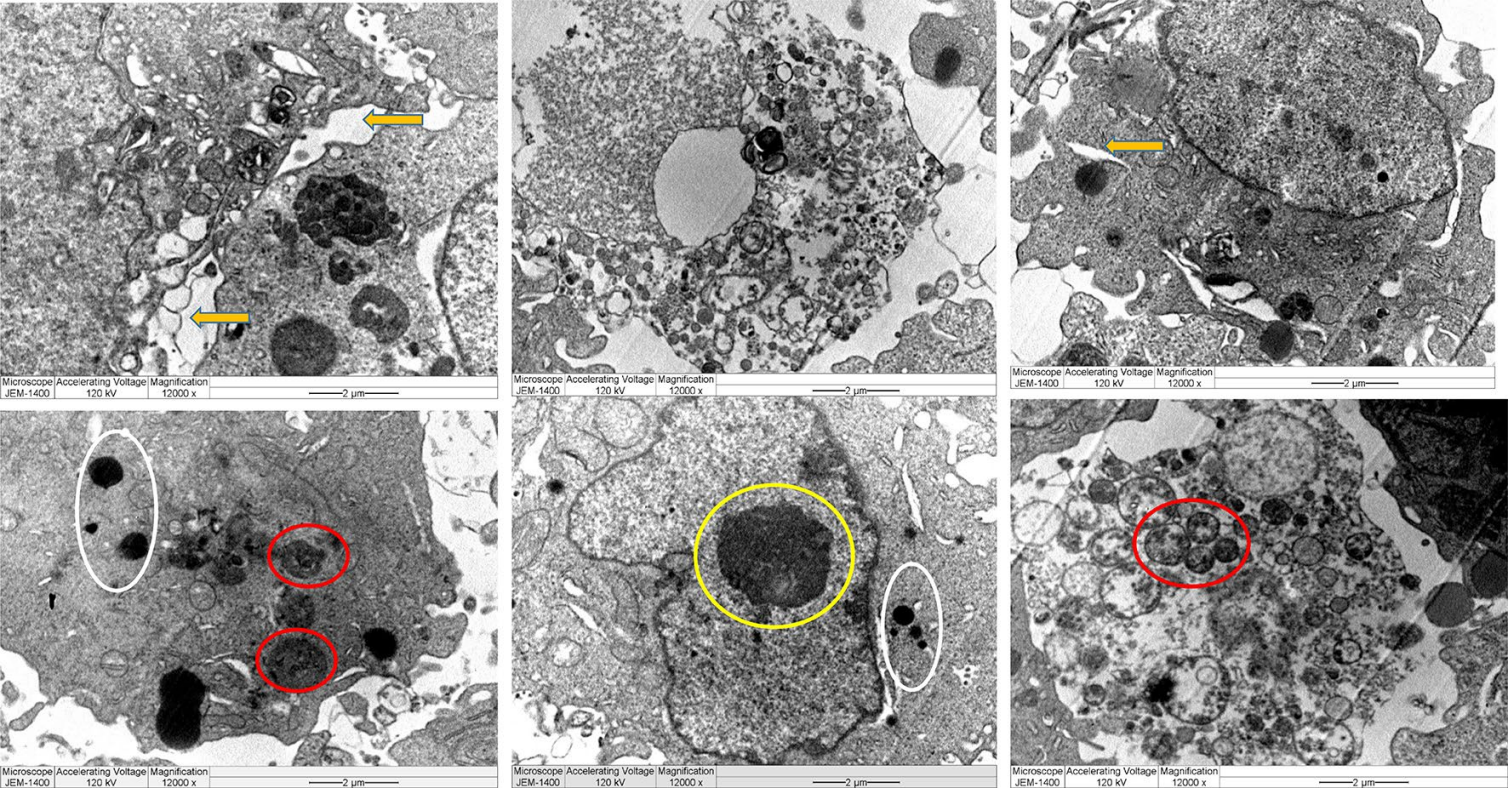
TEM analysis for the human cancer cell lines (MDA MB 23) treated with P-AgNPs at varied magnification displaying ultrastructural alteration displaying over whole cell shrinkage, chromatin condensation (yellow circle) beside peroxisomes (white square), damaged mitochondria (red circle), large vacuoles (yellow arrow) and peroxisome (white circle). Damaged cancerous cells are observed.

**Figure 14:**
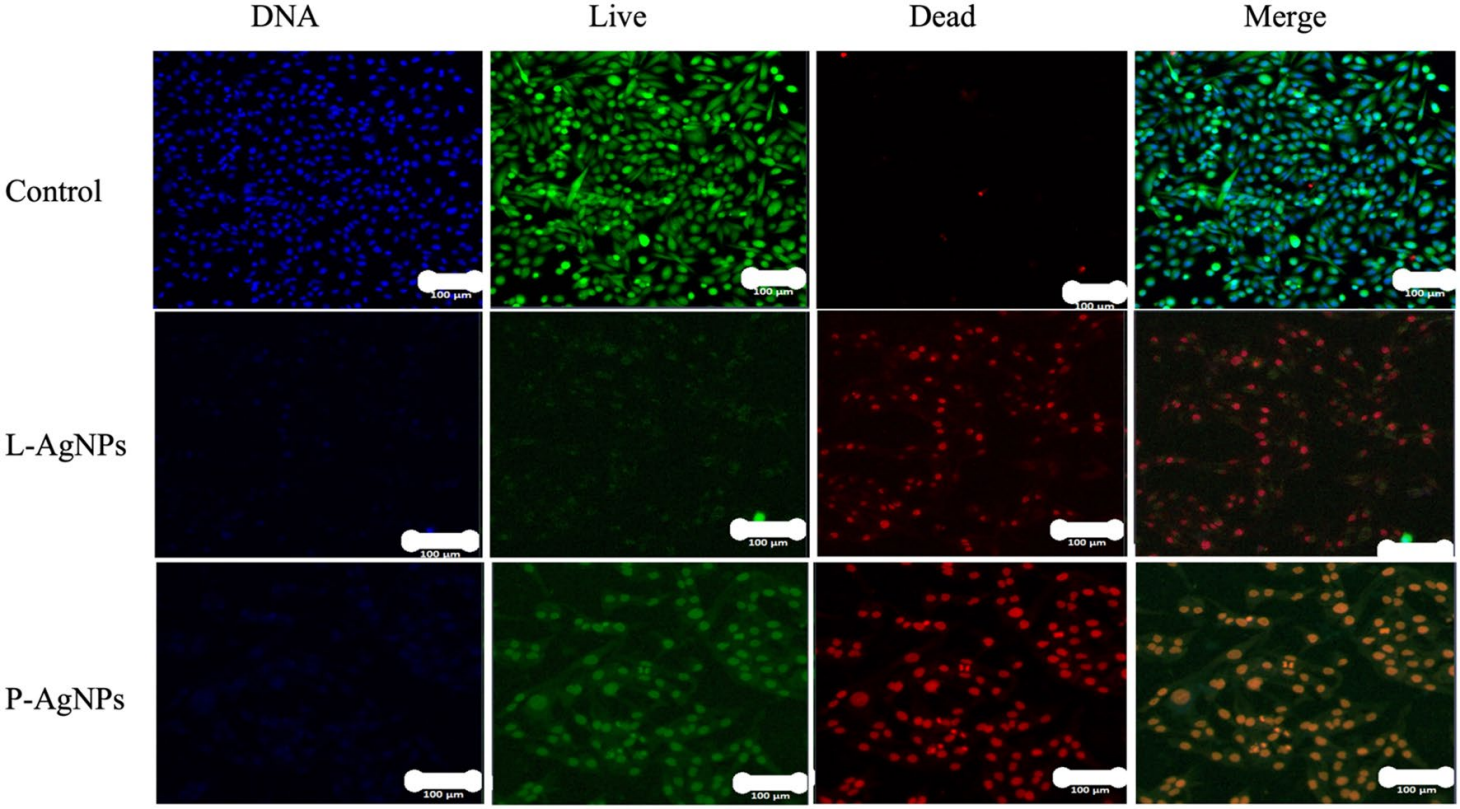
Confocal LSM analysis for MDA MB 231 cell lines treated by L-AgNPs and P-AgNPs beside untreated control. Cells stained with HOECHST33342, blue (DNA) indicated fragmented nuclei, Calcein AM, green (live) showing living cells and those stained by Propidium Iodide, red (dead) showing dead cells besides overview of merged three stains (merge).

The possible mode of action of the biogenic NPs as cytotoxic agents has been investigated using TEM analysis. The cells that suffered high toxicity (MDA MB 231) have been chosen and examined where clear changes in cell structure were noticed (Figs. 12 and 13). Features such as an irregular membrane of the nucleus and shape of the overall cell, low numbers of microvilli, large vacuoles, damaged mitochondria, peroxisomes, chromatin condensation and enlarged mitochondria were reported. LSM was further used for NPs mechanism against treated MDA-MB-231 cells where apoptotic features appeared such as reduced viable cells and stained DNA with red colour PI was noted (Fig. 14).

### Antibacterial effect

**Figure 15:**
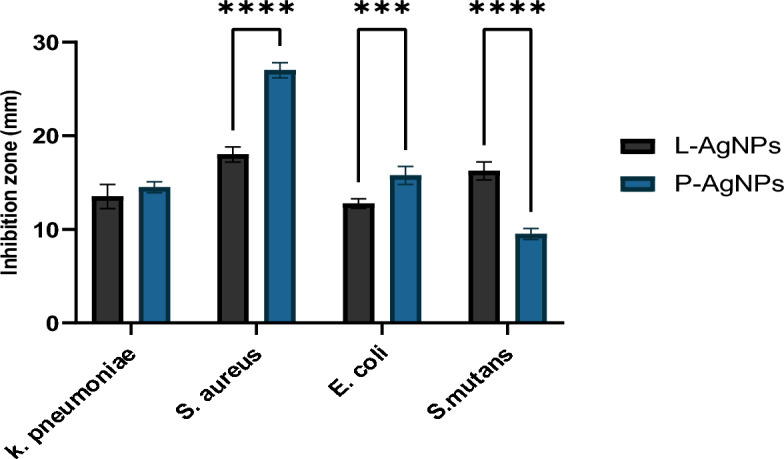
Antibacterial effect of L*-*AgNPs and P-AgNPs against four different bacteria as inhibition zones in mm. Variations between groups were done by One-way ANOVA p < 0.0001 (****) and p < 0.001 (***).

The antibacterial effect of the two seed extracts and NPs prepared by their aid has been investigated against *E. coli*, *K. pneumoniae*, *S. mutans* and *S. aureus* using an agar well diffusion assay. Results are indicated as inhibition zone diameters (mm) as shown in Fig. 15. Seed extract had no antibacterial effect against tested strains but both L-AgNPs and P-AgNPs were efficient. S. *aureus* was the most sensitive strain that showed 27 ± 0.8 and 18 ± 0.8 mm as inhibition zones around the well filled with P-AgNPs and L-AgNPs, respectively (*p* < 0.0001). *S. mutans*; 9.5 ± 0.5 and 16.3 ± 0.9 mm for P-AgNPs and L-AgNPs, respectively (*p* < 0.0001). 15.8 ± 0.9 and 12.8 ± 0.5 mm was the inhibition zone reported in *E. coli* plates for P-AgNPs and L-AgNPs, respectively (*p* < 0.001). *K. pneumonia* indicated 14.5 ± 0.5 and 13.5 ± 1.2 mm for P-AgNPs and L-AgNPs, respectively where no significant variation was noted. The MIC and MBC were determined for the lowest NPs concentrations that inhibit and kill 99% and 99.9% of microbes, respectively. The tolerance level of NPs showed the bactericidal effect of both types of tested NPs (Table 2).

**Table 2:** The MIC and MBC for L-AgNPs and P-AgNPs against tested bacteria.

Microbes	L-AgNPs			P-AgNPs		
	MIC (mg/mL)	MBC (mg/mL)	MIC/MBC	MIC (mg/mL)	MBC (mg/mL)	MIC/MBC
*S. mutans*	0.8	1.1	0.72	0.8	1.1	0.72
*S. aureus*	0.8	1.1	0.72	0.8	1.1	0.72
*K. pneumoniae*	0.8	1.1	0.72	0.8	1.1	0.72
*E. coli*	0.8	1.1	0.72	0.8	1.1	0.72

**Figure 16:**
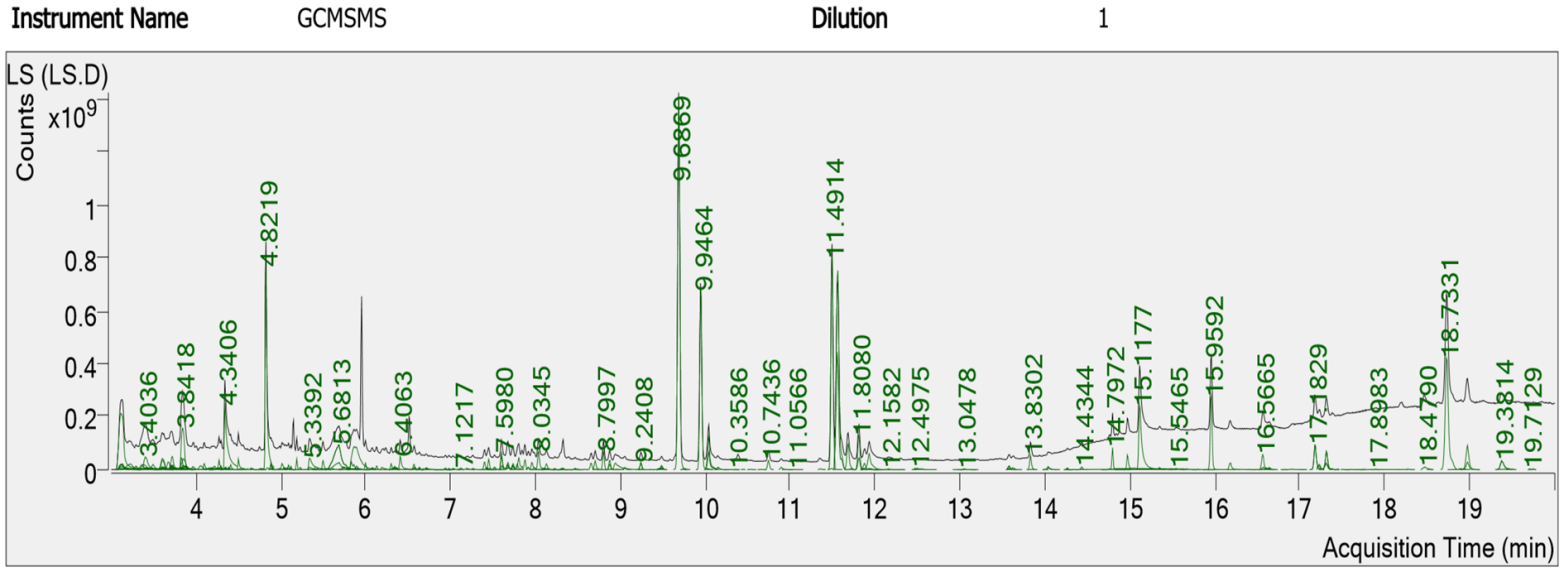
GC–MS analysis of *Lasiurus scindicus* methanolic extract.

**Figure 17:**
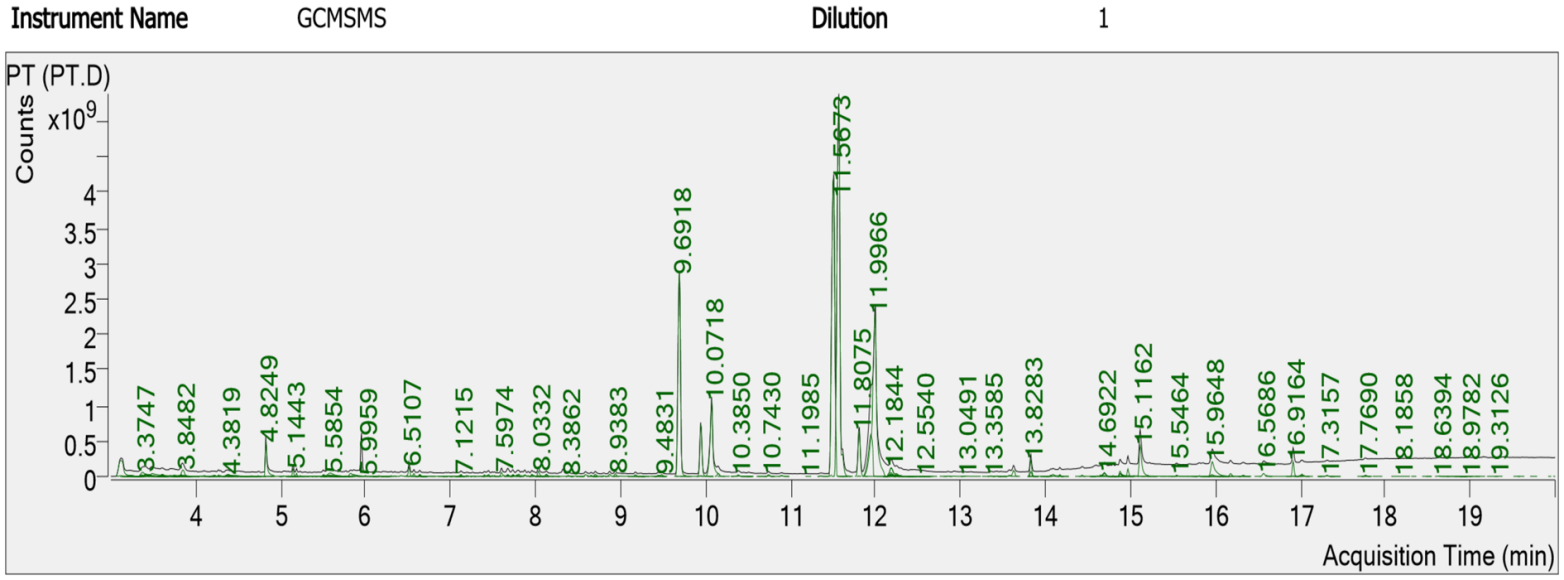
GC–MS analysis of *Panicum turgidum* methanolic extract.

The GC–MS analysis indicated about 200 phytochemicals from *Lasiurus scindicus* (Fig. 16) and another 200 from *Panicum turgidum* extract (Fig. 17). The most abundant compounds were reported in Table 3 (five compounds for *Lasiurus scindicus* methanolic extract) and in Table 4 (six compounds from *Panicum turgidum* methanolic extract).

**Table 3:** The most abundant compounds from the methanolic extract of *Lasiurus scindicus* by GC–MS analysis.

No.	Component RT	Compound name	Formula	Area	Match score
1	4.8219	2-Methoxy-4-vinylphenol	C_9_H_10_O_2_	1,227,375,561	93.9
2	9.6869	Hexadecanoic acid, methyl ester	C_17_H_34_O_2_	2,652,137,934	93.8
3	9.9464	Benzenepropanoic acid, 3,5-bis(1,1dimethylethyl) -4-hydroxy-, methyl ester	C_18_H_28_O_3_	1,207,666,919	90.8
4	11.4914	9,12-Octadecadienoic acid (Z,Z)-, methyl ester	C_19_H_34_O_2_	1,457,131,072	96.2
5	18.7331	Octacosanol	C_28_H_58_O	1,179,238,232	90.0

**Table 4:** The most abundant compounds from the methanolic extract of *Panicum turgidum* by GC–MS analysis.

No.	Component RT	Compound name	Formula	Area	Match score
1	9.6918	Hexadecanoic acid, methyl ester	C_17_H_34_O_2_	6,265,479,133	91.2
2	10.0718	n-Hexadecanoic acid	C_16_H_32_O_2_	2,513,420,748	92.9
3	11.5053	9,12-Octadecadienoic acid (Z,Z)-, methyl ester	C_19_H_34_O_2_	11,079,137,580	94.5
4	11.5673	9-Octadecenoic acid, methyl ester, (E)-	C_19_H_36_O_2_	13,022,238,239	96.1
5	11.8075	Methyl stearate	C_19_H_38_O_2_	1,169,465,495	95.1
6	11.9966	cis-Vaccenic acid	C_18_H_34_O_2_	7,882,145,460	93.7

## Discussion

For their biocompatible nanostructure, biogenic AgNPs can be used for treating cancer and microbes besides varied other potential applications^22^. Characteristics of the NPs are the determining factors for their biological action and activity against varied systems^23^. Current findings observed the biotransformation of Ag^+^ ions into Ag^o^ by increasing the color intensity of the mixture. Plasmon vibration excitation on the AgNPs surface is normally characterized by such color changes^24^. Similar findings were also indicated in our previous studies regarding varied plant extracts^7,25–27^. The analysis of the biogenic L-AgNPs and P-AgNPs indicated nanosized structure and monodisperse NPs since PDI values were below 0.3^28^. Slight variations between L-AgNPs and P-AgNPs were noted in their size and PDI which could be related to the varied biogenic agents used that may involve varied phytochemicals therefore variation in the reduction and stabilization process is expected. *Panicum virgatum* was used for silver nanoparticle formulation^29^ however, no valid study reported the *Lasiurus scindicus* and *Panicum* turgidum as bio mediators in AgNPs formulation. Spherical dispersed NPs with no agglomeration were noted by TEM analysis, such observation could be related to the plant active compounds that cover the NPs and lead to less particle-to-particle adherence^25^. Spherical well-distributed NPs were also obtained when two types of lichen were utilized for their fabrication^15^. Further, the EDX spectrum indicated the occurrence of elemental Ag in the nanoparticle’s solution with another two signals for carbon and oxygen that could partially originate from the phytochemicals of seed extract. Their presence may increase the NPs biocompatibility since originated from biological material and therefore could be good for the biomedical applications. Similar findings have been reported for AgNPs fabricated using plant extracts^30,31^.

FTIR is known as an analytical tool for the detection of inorganic and organic compounds from varied sources^32^. The observed spectrum at 1620–1680 cm^−1^ could be related to C=C (Alkenyl stretch) and those at 3200–3400 cm^−1^ representing the H-bonded OH stretch, Hydroxy group^33^. The slight alteration in the magnitude of the absorption peaks suggests using the seed's biomolecules in NPs fabrication. The biomolecules detected by FTIR and GC–MS analysis could be the main reasons for the reduction and capping of the NPs. Identified compounds from both plant origins were varied according to the GC–MS analysis which might suggest variations in the fabricated NPs characteristics and the biological activities. The small-size monodispersed NPs that were coated with functional groups from both plant seeds were tested for their effect on the metabolic activity of human cell lines (MCF 10A, HCT116, and MDA MB 231). Our results revealed effective NPs in mitigating the tested cells, however, differentially target the HCT116 since a weak effect was noted against MDA MB 231cell lines. Our earlier investigations indicated varied plant extract and their ability in NPs fabrication which showed anti-proliferative and cytotoxic effects^7,25–27^. Green AgNPs as an efficient approach to cure cancer-related diseases is reported recently for NPs prepared by *Artocarpus lakoocha* fruit extract and tested against human prostate adenocarcinoma^34^.

The biogenic NPs mode of action against MDA MB 231cell lines was described as ultrastructural by TEM analysis. Changes might be linked to the high affinity of small size NPs that could enhance NPs-cell interaction and induce their entry leading to cell death. Roy et al.^35^ and Xu et al.^13^ reported various mechanisms for NPs-cell interaction such as oxidative stress, intercellular penetration and cell membrane and wall damage. The damage could also be related to the phytochemicals that cap the NPs and enhance their cell entry due to the expected high compatibility of biogenic NPs to the biological system^36^. An increased number of peroxisomes in NPs-treated cells might indicate the cell response to oxidative damage that led to the production of reactive oxygen species (ROS) since peroxisomes take main part in the metabolism of ROS^37^. ROS as a consequence of NPs against breast cancer has been well-investigated in mediating cell apoptosis^38,39^. No valid report regarding the biological activity of NPs prepared by both tested agents however, *P. turgidum* extract was reported for safe antihepatotoxic activity^4^. Zaki et al.^40^ determined the antifungal and antiprotozoal activities of steroidal saponin from *P. turgidum* extract. Identified compounds could also enhance the extract activity against cancer cell lines tested. The importance of *Lasiurus scindicus* as a medicinal plant was also previously studied^5^. The apoptotic features detected by LSM analysis were previously described by Palvai et al.^41^ and Mohammed and Al-Megrin^25^ when studying colon cancer and MDA-MB-231 cells, respectively. P-AgNPs showed better activity against all tested cell lines compared to L-AgNPs which might be related to their smaller size in average diameter therefore, high activity could be also related to active plant metabolites that may cap the NPs and enhance their activity that detected in the current study by GC–MS. The antibacterial effect of the L-AgNPs and P-AgNPs has been observed. No clear trend of observation was noted against tested microbes concerning bacterial type (Gram stain) and also concerning NPs type. No effect of the seed extract of *P. turgidum* but the antifungal and antiprotozoal ability was previously noted for compounds isolated from *P. turgidum*^40^ which could be related to varied plant part used since the cited study used the arial part but also the material concentration may have an effect. No valid report about the antimicrobial ability of *Lasiurus scindicus*. The nano-antibiotics AgNPs have high potential against diseases from microbes’ biofilms^42^. NPs are known to enhance the production of ROS leading to cell damage and microbial growth suppression^43^. The biological activity of the NPs could be related to the expected higher surface area of the bulk origin therefore great surface activity is obtained^44^. The MIC and MBC were determined for the lowest NPs concentrations that kill 90% and 99.9% of microbes, respectively. The tolerance level of NPs showed the bactericidal effect of both types of the tested NPs since the MBC: MIC is less than 4^16^. The bactericidal effect of AgNPs prepared from various fungal extracts was also recently reported^17^. Recently, Bhat et al.^45^ reported the antimicrobial activity of AgNPs by the leaf extract of *Ixora brachypoda* DC.

## Conclusion

A feasible substitute to chemical drugs against cancer and microbes is an urgent issue since cell drug resistance development is increasing. Our study was designed to find out the ability of *P. turgidum* and *L. scindicus* seed extracts as sources of bio-nanotechnology which is considered as the first report for silver nanoparticles from both sources. NPs prepared from seed extract of *P. turgidum* and *L. scindicus* indicated spherical small-size particles that showed anticancer and antimicrobial efficiency. The ability of both NPs to reduce cell viability was observed however, higher activity was noted against metastatic breast cancer cells compared to colorectal cancer and normal cell lines. The mode of action of biogenic NPs in cell apoptosis has been noted using TEM and LSM techniques where clear ultrastructural changes and damage have been noted. Slight variations in NPs characteristics from different sources and biological activity could be mainly related to seed biomolecules that may take part in NPs preparation which are detected by FTIR and GC–MS analysis. Further investigations are needed to screen the studied NPs on the higher scale of tested cells and microbes.

## Materials and methods

### Plant seeds sources and processing

*L. scindicus* and *P. turgidum* seeds have been obtained from the Royal Commission for Riyadh City (RCRC) in Riyadh, Saudi Arabia nursery during January 2021. The seeds were identified by Dr. Mudawi M. Nour, a researcher in the nursery of RCRC. Experimental research on plants is complied with relevant institutional, national and international guidelines and legislation.

The seeds were cleaned using distilled water then air dried and milled by the milling machine (IKA Werke GMBH and Co., Staufen im Breisgau, Germany) into a fine powder. Plastic bags were used for seed preservation and kept at room temperature until further analysis.

### Seed extracts and AgNPs fabrication

The seeds' aqueous extracts were made using individual seed powder (2g) which was added to 100 mL distilled water. The mixture was incubated for 20 min at 90 °C water bath thereafter, mixtures were filtered by Whatman No. 1 filter paper. Subsequently, 10 mL from each filtrate was added to 90 mL of AgNO_3_ (1 mM) and re-heated at 90 °C for 10 min. The reaction medium has been kept at room temperature in the dark condition and color transformation has been noticed to attain a stable color. Then, the reaction medium was centrifuged at 13,000 rpm for 20 min and the pellet was rinsed two times using distilled water at the same centrifugation states and then kept at room temperature for drying. Lastly, from each NPs, a concentration of 1 mg/mL has been prepared for further analysis.

### NPs properties

Biogenic NPs were described using various techniques. A spectrophotometer (BIOCHROM Libra S60PC, Serial Number: 119377, England) was used to measure the ultraviolet–visible (UV–Vis) Spectroscopy absorption for the prepared NPs. The analysis of hydrodynamic size was investigated by a Zetasizer (NANO ZSP, Serial Number: MAL1034318, ver 7.11, Malvern Instruments Ltd., Malvern, UK) using a dynamic light scattering (DLS) system. Electron transmission microscopy (TEM) was used for morphology and size distribution employing a TEM system at 80 kV voltage (JEM-1011, JEOL, Tokyo, Japan). A scanning electron microscope (JEOL JED-2200 series) was used for energy-dispersive X-ray spectroscopy (EDS) to investigate the surface of the NPs and detect the existence of elemental Ag.

### Evaluation of surface functional groups

Seed extracts and the prepared NP solutions were tested by Fourier-transform infrared spectroscopy (FTIR) analysis to find out the probable biomolecules and organic agents present. Such an approach was achieved by an FTIR spectrometer (SPECTRUM100, PerkinElmer, Wellesley, USA), utilizing a diffuse reflectance attachment at 450–3500 cm^-146^.

### Antitumor activity enhanced by biosynthesized NPs

The effect of biogenic NPs on MCF 10A cells as a standard cell line, the colorectal cancer cell line (HCT116) and the breast cancer cell line (MDA MBA 231) viability has been evaluated by an MTT test. Cell cytotoxic effect analysis was done against standard cell line MCF 10A (ATCC-CRL-10317) and MDA MBA 231 (breast cancer cell lines) that originated from from a pleural effusion of a 51-year-old caucasian female with a metastatic mammary adenocarcinoma^45^ and HCT116 (human colorectal carcinoma cell line isolated from an adult male)^46^. The work was done in King Abdullah International Medical Research Center (KAIMRC) and they provided the cell lines. At 96-well plate cells were cultured (5 × 10^4^ cells/well) at 95%/5% (humidified air/CO_2_) at 37 °C and. 24 h after, media was discarded and phenol-red free DMEM involving has been added in addition to 0.5% fetal bovine serum (FBS). Afterward, tested cell lines were subjected to varied concentrations of NPs in the range of 0–360 μg/mL for L-AgNPs and 0–288 μg/mL for P-AgNPs for 48 h. Then the media was discarded and PBS was used for cell washing. A spectra max microplate absorbance reader (Molecular Devices, San Jose, CA, USA) has been applied for MTT evaluation at 570 nm absorbance^25^. Moreover, for apoptosis evaluation, TEM and LSM analysis have been employed to investigate NPs treated MDA MB 231 cell lines.

### Transmission electron microscopy for ultrastructural cell changes

NPs-treated MDA-MB-231 cells and the untreated control were investigated using TEM analysis. Cell sections have been tested according to Ali et al.^47^ with minor changes, then sections were loaded on a grid (Product G200-Cu, EMS, Ottawa, Ontario, CA) and 1% uranyl acetate (Product 93-2840, STREM CHEMICALS, Newburyport, MA, USA) was added for staining in dark conditions for 15 min. afterward normal saline has been applied six times for washing, then 0.5% lead citrate (Product 17810, EMS) was applied. After drying, a TEM system (JEOL JEM 1400) was used for sample evaluation.

### Laser scanning microscopy analysis

A dish involving eight wells (Ibidi, Munich, Germany) has been used for loading the MDA-MB-231 cells before 24 h of the treatment. Afterward, cells were exposed to 16 μg/mL and 20 μg/mL from L-AgNPs and P-AgNPs respectively then kept for 24 h at 37 °C under CO_2_ (5%). Consequently, PI (Red), HOECHST 33,342 (Blue) and nucleus Calcein AM (Green) staining have been used for dead cells, nuclei and live cells, respectively. Imaging was noted by the LSM780 microscope system (Zeiss, Jena, Germany) using an argon laser at 488 nm/530 nm for Calcein AM, an Intune laser at 90 nm/640 nm for PI and a UV laser diode at 350 nm/460 nm for HOECST 33342.

### Antibacterial susceptibility test

Well-diffusion agar plates have been applied to assess the inhibitory potential of the tested seed extracts and fabricated NPs. *Streptococcus mutans, Staphylococcus aureus*, *Escherichia coli* and *Klebsiella pneumoniae* (Bio-house medical lab, Riyadh, Saudi Arabia) have been tested against L-AgNPs and P-AgNPs. Suspensions of 0.5 McFarland concentration (1.5 × 10^8^ CFU/mL) for each strain were prepared in saline by direct colony suspension method. Afterward, agar plates were used for bacteria cultures and wells were made for the addition of the tested agents (40 µL) at 1 mg/mL concentration. NPs were added separately to individual wells and maintained for 1 h under aseptic conditions for drying. then plates have been incubated for 24 h at 37 °C. Afterward, the inhibition zone in mm around each well was evaluated. Distilled water has been applied as a negative control.

### Minimum bactericidal and inhibitory concentrations (MBC and MIC)

Biogenic NPs have been assessed for their inhibitory effect using the serial dilution approach. About 100 μL of each bacterial suspension at a concentration of 0.5 McFarland were applied in 96-well plates at varied concentrations of both biogenic L-AgNPs and P-AgNPs (0.2, 0.5, 0.8, 1.1, 1.4, 1.7, 2.0 and 2.3 mg/mL) and incubated at 37 °C for 24 h. The agar well diffusion approach was used for MBC and MIC values determination.

### The phytochemicals identification by GC–MS analysis

*Lasiurus scindicus* and *Panicum turgidum* were screened using an Agilent GC–MS system (Agilent Technologies 7890B Gas Detector and Agilent Technologies 7000D Mass Spectrometer Detector, Santa Clara, CA, USA) with helium (high purity). The extraction methods were done by adding 10 g of the plant powder to 100 mL methanol and kept overnight then filtered and the supernatant was used for the GC–MS and the conditions were set according to Olivia et al.^48^. at thesplitless mode, the injection volume was 10 μL and the injector temperature was 280 °C. The detected single molecule was identified using the libraries of the National Institute of Standards and Technology (NIST08) and WILEY libraries depending on mass spectra and relative indices.

### Statistical analysis

Data for three replicates are given as mean ± standard deviation (SD). Differences amongst considered parameters have been statistically achieved using the Prism 9.1 software (GraphPad Software Inc., La Jolla, CA, USA) by one-way analysis of variance (ANOVA). *p* < 0.0001, *p* = 0.001 and *p* < 0.01 are indicating significance of the data. Graphs have been prepared using the same software. The values for the IC_50_ for the tested agents against each cell line have been assessed by Log viability vs. normalized response–variable slope.

## Data Availability

The datasets used during the current study are available from the corresponding author upon reasonable request.
