# Author Correction: Bioactive silver nanoparticles fabricated using *Lasiurus scindicus* and *Panicum turgidum* seed extracts: anticancer and antibacterial efficiency

**DOI:** 10.1038/s41598-024-70963-w

**Published:** 2024-09-12

**Authors:** Najla Alburae, Rahma Alshamrani, Afrah E. Mohammed

**Affiliations:** 1 Department of Biological Sciences, King Abdulaziz University, P.O. BOX 80206, 21589 Jeddah, Saudi Arabia; 2 Department of Biology, College of Science, Princess Nourah Bint Abdulrahman University, P.O. Box 84428, 11671 Riyadh, Saudi Arabia

Correction to: *Scientific Reports* 10.1038/s41598-024-54449-3, published online 20 February 2024

The original version of this Article contained an error in the Acknowledgements section. The digits of the Grant Code were incorrect.

“This research work was funded by Institutional Fund Projects under Grant No. (IFPHI038-247-2020). Therefore, the authors gratefully acknowledge technical and financial support from the Ministry of Education and King Abdulaziz University, DSR, Jeddah, Saudi Arabia.”

now reads:

“This research work was funded by Institutional Fund Projects under Grant No. (IFPHI036-247-2020). Therefore, the authors gratefully acknowledge technical and financial support from the Ministry of Education and King Abdulaziz University, DSR, Jeddah, Saudi Arabia.”

The original Article has been corrected.

